# The effect of priority setting decisions for new cancer drugs on medical oncologists' practice in Ontario: a qualitative study

**DOI:** 10.1186/1472-6963-7-193

**Published:** 2007-11-28

**Authors:** Scott R Berry, Stacey Hubay, Hagit Soibelman, Douglas K Martin

**Affiliations:** 1 Division of Medical Oncology/Hematology, Sunnybrook Health Sciences Centre, Department of Medicine, University of Toronto, Toronto, Canada; 2 University of Toronto Joint Centre for Bioethics, Toronto, Canada; 3 Grand River Regional Cancer Centre, Division of Medical Oncology, Kitchener, Ontario, Canada; 4 Student, Faculty of Nursing, University of Toronto, Toronto, Canada; 5 Department of Health Policy, Management and Evaluation, University of Toronto, Toronto, Canada

## Abstract

**Background:**

Health care policies, including drug-funding policies, influence physician practice. Funding policies are especially important in the area of cancer care since cancer is a leading cause of death that is responsible for a significant level of health care expenditures. Recognizing the rising cost of cancer therapies, Cancer Care Ontario (CCO) established a funding process to provide access to new, effective agents through a "New Drug Funding Program" (NDFP). The purpose of this study is to describe oncologists' perceptions of the impact of NDFP priority setting decisions on their practice.

**Methods:**

This is a qualitative study involving semi-structured, in-depth interviews with 46 medical oncologists in Ontario. Oncologists were asked to describe the impact of CCO's NDFP drug funding decisions on their practice. Analysis of interview transcripts commenced with data collection.

**Results:**

Our key finding is that many of the medical oncologists who participated in this study did not accept limits when policy decisions limit access to cancer drugs they feel would benefit their patients. Moreover, overcoming those limits had a significant impact on oncologists' practice in terms of how they spend their time and energy and their relationship with patients.

**Conclusion:**

When priority setting decisions limit access to cancer medications, many oncologists' efforts to overcome those limits have a significant impact on their practice. Policy makers need to seriously consider the implications of their decisions on physicians, who may go to considerable effort to circumvent their policies in the name of patient advocacy.

## Background

Physician practice is a key determinant of health care outcomes, expenditures and quality of care[1,2]. Health care policies, including drug-funding policies, have a major influence on physician practice [1]. However, to our knowledge, there are no studies of the effect of drug funding policies on oncologist practice. Funding policies are especially important in the area of cancer care for several reasons. Cancer is one of the leading causes of death in Canada. It is estimated that there will be 153,100 new cases of cancer and 70,400 cancer deaths in Canada in 2006[3]. Beyond its human toll, cancer accounts for 8.3% of health care expenditures in Canada[4]. A 1997 study estimated that through direct and indirect expenditures, cancer cost the Canadian economy 13 billion Canadian dollars in 1993[4]. The practice of oncologists therefore has a significant effect on the health of Canadians and health care expenditures.

In recognition of the rising costs of new cancer drugs, CCO's NDFP was established in 1995 to cover the cost of certain new and expensive IV cancer drugs. The NDFP worked in concert with the CCO Practice Guideline Initiative (CCOPGI), which involves a formal and rigorous process of evidence review, knowledge synthesis and consensus development by medical oncology content and methodology experts in Ontario[5]. Until 2004 (and at the time of this study), a "Policy Advisory Committee" (consisting of administrators, oncologists, oncology researchers, a pharmacist, an ethicist, patients, and members of the public) made funding recommendations to the NDFP based on a rigorous review of practice guidelines from the CCOPGI and their assessment of the quality of evidence, benefits observed and costs of the new medications[6].

The specific goal of the NDFP was to ensure equitable access for patients to newly available systemic cancer treatments that have been proven to be effective for their condition as stated in CCO Practice Guidelines. The program reimburses hospitals and cancer centres for drugs administered to patients who meet specific eligibility criteria. This program has grown from a $7 million Canadian dollars program funding 6 drugs for 2354 patients in 1997/98 to a $64 million Canadian dollars program funding 16 drugs for over 14,000 patients in 2003/04. Although the process and rationales used for these decisions have been studied in detail [6-8], the impact of these policies on medical oncologists' practice has not been studied. The purpose of this study is to describe medical oncologists' perceptions of how priority setting decisions for new cancer drugs affected their practice.

## Methods

### Design

This was a qualitative study involving in-depth interviews with medical oncologists in Ontario in 2002/2003.

### Participants/setting

Medical oncologists from across Ontario in a variety of practice settings. There were approximately 140 medical oncologists in Ontario at the time of the study. Medical oncologists were identified from publicly available sources (College of Physicians and Surgeons of Ontario website and the Canadian Medical Directory). Purposive sampling targeted oncologists who had been practicing for at least 5 years were so that they were able to comment on their practice before and after the advent of the NDFP. Participants were identified from a variety of practice settings: i.e. comprehensive cancer centres, general hospitals and from both small and large centres.

### Data collection

In-depth, semi-structured, open-ended interviews were conducted with 46 oncologists (for demographic information see Table 1), in person or by phone, and were audiotaped and transcribed. Participants were asked to describe how CCO's NDFP decisions have affected their practice. The interviewers pursued and clarified information about emerging themes. Interviews continued until themes were "saturated" (ie no further new concepts were emerging from interviews.). An initial interview guide was developed (appendix 1) and, as is typical in qualitative research, was revised during the study to explore emerging themes.

**Table 1:** Demographic Information

**Sex**		
	Female	10
	Male	36
**Years Practicing Oncology**		
	> 30	4
	>20	11
	>10	24
	5–9	7
**Country Where Graduated From Medical School**		
	Canada	39
	Europe	4
	Asia	3
**Practice Details**		
	Group Practice	39
	Solo Practice	7
	Comprehensive Cancer Centre	25
	Other	21
	University Hospital	24
	Community Hospital	22
**Involvement in CCO Practice Guideline Initiative**		
	Participant	18
	Non-Participant	25
	Unknown	3
**Region (By Ontario local health integration network)**		
	Toronto Central	14
	Central East	7
	Mississauga Halton	6
	Champlain (Ottawa Region)	4
	South West (London Region)	3
	Hamilton Niagara	3
	South East (Kingston Region)	2
	Central West	2
	Central	2
	Waterloo Wellington	1
	North Simcoe Muskoka	1
	North East	1

### Data analysis

Data analysis proceeded concurrently with data collection. Interview transcripts were read and participants' views regarding the impact of drug funding decisions on their practice were identified and coded. Coded units were labelled as specific concepts relating to impact on practice. Codes were continuously compared within and between transcripts to ensure consistency and comprehensiveness. Similar concepts were grouped together under overarching themes and the data were recoded by theme. The themes were then organized according to perceived importance, which was based on both prevalence and the participants' emphasis. Finally, descriptions of the themes were developed using the participants' own words.

The trustworthiness of our findings was enhanced in three ways. First, three investigators coded the raw data to ensure the authenticity of the coding scheme; the final coding scheme was developed by consensus and used for the analysis. Second, another investigator familiar with the data participated in developing the interpretation. Third, a draft of this paper was endorsed by participants in a "member check."

### Ethics

This study was approved by the Research Ethics Board at Sunnybrook and Women's College Health Sciences Centre. All participants provided informed consent for the interview.

## Results

Our key finding is that many of the medical oncologists who participated in this study did not accept limits when policy decisions limit access to cancer drugs they feel would benefit their patients. Moreover, overcoming those limits had a significant impact on oncologists' practice in terms of how they spend their time and energy and their relationship with patients. In the end, however, the perception of most oncologists is that they get what they think will maintain the quality of patient care.

Five key themes emerged from the analysis including: 1. Access to medications, 2. Overcoming limits to access, 3. Impact on the physician-patient relationship, 4. Disengagement of clinicians from priority setting process and 5. Quality of care. Verbatim quotes from participants are included for illustration.

### Access to medications

Participants acknowledged that the NDFP had a rigorous process and had improved the evaluation of cancer drugs and access to drugs approved by the process:

*[The NDFP] has been a great help in terms of treatment of cancer patients... It incorporates some of the practice guidelines into place so that people are not using drugs at random that may be very expensive. It has also provided me the opportunity to ... provide these patients with some of the expensive drugs which otherwise our pharmacy would not be able to...So I think it has actually, in general, improved ... the quality of care ... that we are able to provide to our patients*.

However they also expressed significant concern about limits on access to drugs. Some oncologists were concerned that the limitations imposed within the funding guidelines did not allow them the flexibility required to make treatment decisions for individual patients. Others felt that there was often a significant time-lag between demonstration of a drug's efficacy and a funding decision by the NDFP, although participants felt that this was improving over time:

*And so – and in many ways it's restricted our ability to use these drugs more than existed before the program existed, ... I find it very slow ... certain categories of patients that can be well defined. But there are also patients who don't fit into the normal situations ... The way the CCO program is, it has very precise indications for drugs. And patients who fall outside those precise indications, tough*.

### Overcoming limits to access

When the NDFP limited access to what they felt would be effective medications for their patients, participants generally found a way to get the medications they wanted for their patients. However, overcoming the limits and getting these medications required significant stress and time. The following quotation illustrates this "hassle factor":

*...We call up the drug company. We spoke to _____ Hospital. We spoke to CCO. I spoke to _______ of the ______ Disease Site Group. We then called the company; we called two MPP's, and we called the third-party insurer from one of the patients who had drug coverage*.

If the NDFP would not fund the drug they wanted for their patients, many physicians would spend considerable time and effort finding an alternate funding source for the drug. The alternate funding sources were quite varied and included enrolling a patient on a clinical trial if they were eligible, getting the local hospital to pay for the drug, appealing to another government funding program for non-IV drugs, looking for support from local charities, using leftover drug supplies from patients who had procured funding for a drug and "gaming" the system or lying on forms so that patients would fit funding criteria for a drug.

One of the participants explained:

*...you have to put the code in why this patient should get funding from the government for this drug, and they don't always fit the criteria so you wind up lying...because you want to help your patients*.

These activities were time-consuming and contributed to the physicians' stress. To reduce the time spent on these matters, some participants would delegate tasks associated with seeking alternate funding sources to administrative assistants, nurses, pharmacists or social workers.

### Impact on physician-patient relationship

Priority setting decisions had a significant impact on discussions oncologists had with their patients, including the time spent on discussions with patients, moral distress regarding those discussions with patients, as well as forcing uncomfortable discussions with patients. When a drug was funded by the NDFP, participants felt that less discussion was necessary but there was still significant time needed to discuss drugs where access was limited.

Many participants reported that funding issues led to uncomfortable discussions with patients, including angry responses from patients who were told that certain treatment options were not available due to funding constraints. Concern was expressed regarding how these discussions would impact patient trust. One participant explained:

*Once in a while a patient might feel uncomfortable and they don't come back to see me any more and then remain in the centre and most of our colleagues in the centre are quite understanding*.

Situations where a particular drug was not funded created moral distress for many participants. Some felt it was important to avoid mentioning funding issues in order to protect patients from added anxiety while others felt that fully informing patients meant that they must explain that certain treatments were not funded.

### Disengagement from the funding processes

Participants reported that they did not feel engaged in the NDFP decision-making process because of the perceived inflexibility of some funding guidelines, the time and stress associated seeking alternate funding sources and lack of a formal appeals mechanism.

### Quality of care

Most participants did not perceive the NDFP as affecting overall quality of care. Either drugs were funded by the NDFP or physicians overcame limits to drugs not funded by the NDFP. One oncologist's response summarizes this sentiment:

*We would have provided the drugs regardless of the funding. So it doesn't make any difference to the pattern of practice we have. It helps balance the hospital budget*.

However, this perception was not universal. Some participants acknowledged not being able to access medications with "level 1" evidence. One participant pointed out:

*the funding process as dictated by guidelines restricts drugs to a certain group of people. Some oncologists may choose to find access through different means, some not ... it takes a huge amount of time to try and find access through different means thus taking time away from our patients*.

## Discussion

While previous studies have described funding policy development for new and expensive cancer drugs, this is the first study to describe oncologists' perceptions of the impact of those policies on oncologists' practice. We found that many oncologists do not accept limits to drugs they believe to be necessary for their patients, even when the decisions are made by a priority setting committee specifically appointed to make funding policy decisions. Time and energy spent overcoming those limits have a substantial impact on their practice. In the end, however, most oncologists perceive that the quality of care they provide for their patients is maintained – because they do what they can to get the drugs that are not funded.

Three previous studies have described priority setting for cancer drugs, focusing on the decision making process and reasoning used by priority setting bodies [6-8]. While these studies provide important information on *how *priority setting decisions are made, our study adds important information about how the priority setting policies that are developed affect physician practice.

Oncologists in our study did not appear to accept limits placed on them by funding policy decisions. One possible reason for this is that they did not accept these limits as being legitimate or fair. Daniels has proposed that limits would be accepted if guided by a process that highlights, "a transparency about the grounds for decisions; appeals to rationales that all can accept as relevant to meeting health needs fairly ... and that connect well with the goals of various stakeholders in the many institutional settings where these decisions are made[9]." The participants in this study did not feel engaged with the process used to make priority setting decisions and this may be a major reason for not accepting limits. A previous survey of Canadian oncologists reported that more than 80% of respondents agreed that clinical practice guidelines were good educational tools, convenient sources of advice, and intended to improve quality of care; only 42% of the oncologists surveyed felt that clinical practice guidelines were intended to cut costs [10]. Our study suggests that despite the positive attitudes towards practice guidelines, which were integral to the NDFP's funding decisions[5], efforts to improve oncologists' engagement in funding policy decisions by the NDFP are required. One initial, concrete step to improve engagement with clinicians would be ensuring that they know as much as possible about the decision-making processes used for cancer drug funding decisions. Two previous papers[6,7] have described the strengths of those processes but Singer's[7] study reports that the reasoning behind those decisions was not widely publicised outside the committee. Better publicity about how decisions are made might not only help engage oncologists but also help oncologists understand that the decision makers are sometimes restricted by the evidence based guidelines.

On the other hand, some oncologists may be unwilling to accept any limits on care, no matter how fair the process. Many of the participants in this study were willing to do whatever was necessary to obtain funding for a drug they felt would be beneficial for their patients. It may be that one of the primary tenets of priority setting – that limits must be set to meet population health needs in the face of resource constraints – will not be easily accepted by physicians who see themselves solely as patient advocates [11]. Policy makers will need to consider how they will deal with physicians who will not accept limits to accessing drugs even if set by an "ideal" decision making body.

Three findings from this study may conflict and therefore provide a useful starting point for further exploration. First, at least some oncologists believe that at least some priority setting decisions are limiting access to drugs that are necessary for quality care. Second, many oncologists appear to be spending a significant amount of time and energy on administration and negotiation related to accessing drugs in situations where access has been limited by policy makers. Third, most oncologists in this study claimed that none of this affected the quality of patient care. This begs several questions: When oncologists want access to drugs that policy makers have restricted, are these oncologists right or wrong? Are 'medically necessary' drugs being restricted and, if so, is that wrong? Should oncologists be spending their time and energy on getting access to restricted drugs or should this time and energy be directed to patient care? If they spent more time on patient care would this result in more patients being seen and, perhaps, reduced waiting lists? Certainly, further information from studies accurately quantifying how much time is spent on activities to access unfunded drugs would be useful in informing debates generated by the last two questions.

According to our findings, priority setting decisions had a significant impact on physician-patient relationships. Communicating with patients effectively about difficult subjects is important to maintain an effective and productive physician-patient relationship. Significant work has been done to help improve communication about other difficult topics like end-of-life care [12-14]. There are proposed frameworks [15] on how to communicate effectively about priority setting decisions and further work in this area is warranted.

There are two major limitations to this study. First, since this study described the views of a strategically selected group of oncologists about their practices in response to a specific priority-setting process at a given time, the findings may not be generalizable to other physicians in different practice settings. As priority-setting processes evolve (as they have in Ontario, where there is now a greater influence of cost-effectiveness data), further research will be needed to assess how physicians' practice is affected by these processes. Second, the physicians in this study shared their *perceptions *of how priority setting decisions affected their practice – this may not coincide with their actual practice. However, it is likely that lessons gleaned from this study, and the questions it raises, can help priority setting decision makers, and clinicians who must practice in the aftermath of their decisions.

## Conclusion

In conclusion, this study has shown that when priority setting decisions limit access to cancer medications, oncologists' effort to overcome those limits have a significant impact on their practice. Policy makers need to seriously consider the implications of their decisions on physicians, who may go to considerable effort to circumvent their policies in the name of patient advocacy.

## Competing interests

The authors declare that they have no financial competing interests

Dr Berry was a member of the Cancer Care Ontario Policy Advisory Committee

## Authors' contributions

**Scott Berry **participated in the conception and design of the study, analysis and interpretation of data and drafting and revising the article critically for important intellectual content

**Stacey Hubay **participated in the conception and design of the study, acquisition, analysis and interpretation of data and revising the article critically for important intellectual content

**Hagit Soibelman **participated in acquisition, analysis and interpretation of data and revising the article critically for important intellectual content

**Douglas Martin **participated in the conception and design of the study, analysis and interpretation of data and drafting and revising the article critically for important intellectual content

All authors read and approved the final manuscript

## Appendix 1

### Interview Guide

*Thank-you for agreeing to be interviewed for this study. The purpose of this study is to describe the impact of Cancer Care Ontario's New Drug Funding Program decisions on your practice. All responses will be kept completely confidential*.

*As you know, there are many new and expensive chemotherapies available for cancer patients. Cancer Care Ontario has a funding program for new drugs that will pay for some of these drugs for certain indications*.

1. Tell me about how Cancer Care Ontario's New Drug Funding Program has effected the quality of care you provide for your patients

2. Describe the effect Cancer Care Ontario's New Drug Funding Program has had on your decisions to prescribe new cancer chemotherapies.

3. Tell me about how have you dealt with situations where you wanted to prescribe a drug for a patient, but the drug was not funded. How has this changed since the New Drug Funding Program started funding new drugs?

4. Describe the effect of either Cancer Care Ontario's New Drug Funding Program on discussion of chemotherapy treatment options with patients.

5. Tell me about the decisions of the New Drug Funding Program that have had the biggest impact on your practice.

## Pre-publication history

The pre-publication history for this paper can be accessed here:


